# Supplementary material for: Individual Immune Response to SARS-CoV-2 Infection—The Role of Seasonal Coronaviruses and Human Leukocyte Antigen
Source: Biology (Basel). 2023 Sep 28;12(10):1293. doi: 10.3390/biology12101293 (PMC10603889; doi:10.3390/biology12101293)
Supplement: Supplementary file 1 [file biology-12-01293-s001.zip › biology-2614360-supplementary.pdf]

biology MDPI

Individual Immune Response to SARS-CoV-2 Infection—The Role of Seasonal Coronaviruses and HLA

Karla Rottmayer, Henry Loeffler-Wirth, Thomas Gruenewald, Ilias Doxiadis and Claudia Lehmann

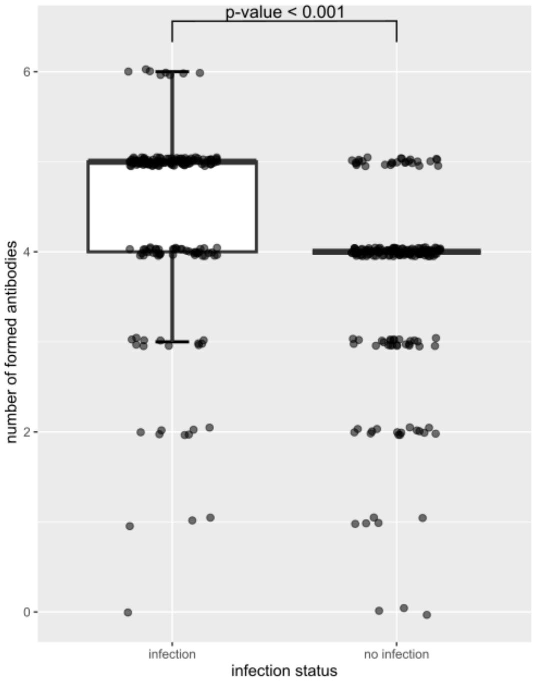

Figure S1. Number of antibodies exceeding the cut-offs that are formed against the sCoVs (hCoV-229E, hCoV-HKU1, hCoV-NL63, hCoV-OC43), MERS-CoV or SARS-CoV. The number of detectable antibodies against the seasonal coronaviruses differed between the infected and non-infected group (p-value < 0.001 in Wilcoxon rank-sum test) In the group of persons with SARS-CoV-2 infection, a median of five antibodies were detectable (left boxplot). Among the uninfected individuals, the median was four (right boxplot).

Detailed results to subsection 3.2.

3.2.1. Response to SARS-CoV-2 after infection or vaccination

This analysis examines the antibody formation as response to the first immunization event. 266 individuals who were first infected and 12 individuals who received their first vaccination were included. We find that more than 80% of individuals in the SARS-CoV-2 infected group had detectable antibodies against the full spike protein, the receptor binding domain, and the S2 domain (Figure S2). Antibodies to the S1 domain were found in 75% of the infected individuals. The lowest response in this group was to the nucleocapsid protein with 63%, which is in line with our findings above. The vaccinated group showed similar responses with more than 75% having detectable antibodies against the whole spike protein, the S1, RBD, and S2 domains, however no individual in the vaccinated group had detectable antibodies against the nucleocapsid protein (Figure S2).

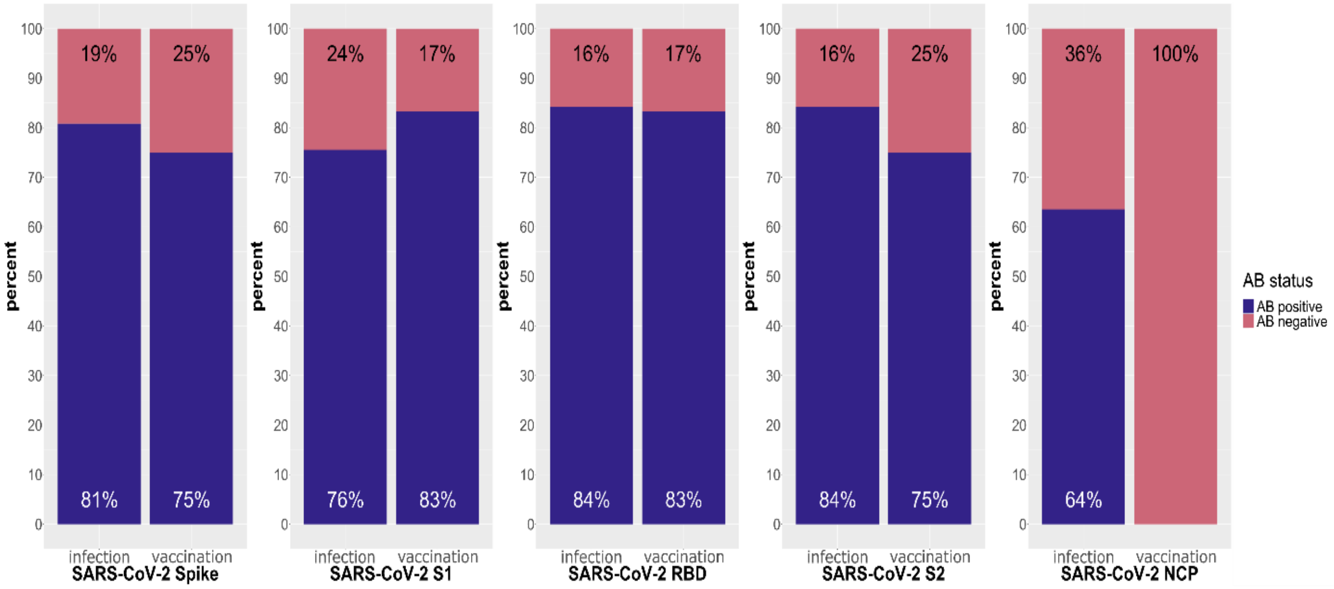

Figure S2. Antibody formation against SARS-CoV-2 domains full spike, S1, RBD, S2, and nucleocapsid protein in the SARS-CoV-2 infected or vaccinated groups. The left columns represent individuals with prior SARS-CoV-2 infection and the right columns represent individuals with vaccination as their first immunizing event, respectively. The blue bars indicate proportions of individuals with a positive antibody response, the rose bars indicate antibody response below the cut-offs.

Both groups, subjects infected with SARS-CoV-2 and those who were vaccinated, developed specific antibodies to each SARS-CoV-2 antigen with an abundance of more than 2 IU/ml, except for the nucleocapsid protein (Figure S3). In particular, we see very similar antibody levels in both groups for all antigens, except for the nucleocapsid protein, where antibodies are only formed in those individuals who have been infected. Thereby, NCP antibody levels are comparable to the other domains with abundances distributed around 2 IU/ml. The qualitative analysis shown in Figure 8 is similar to quantitative results obtained by transformation of the results into IU/ml (Figure S3).

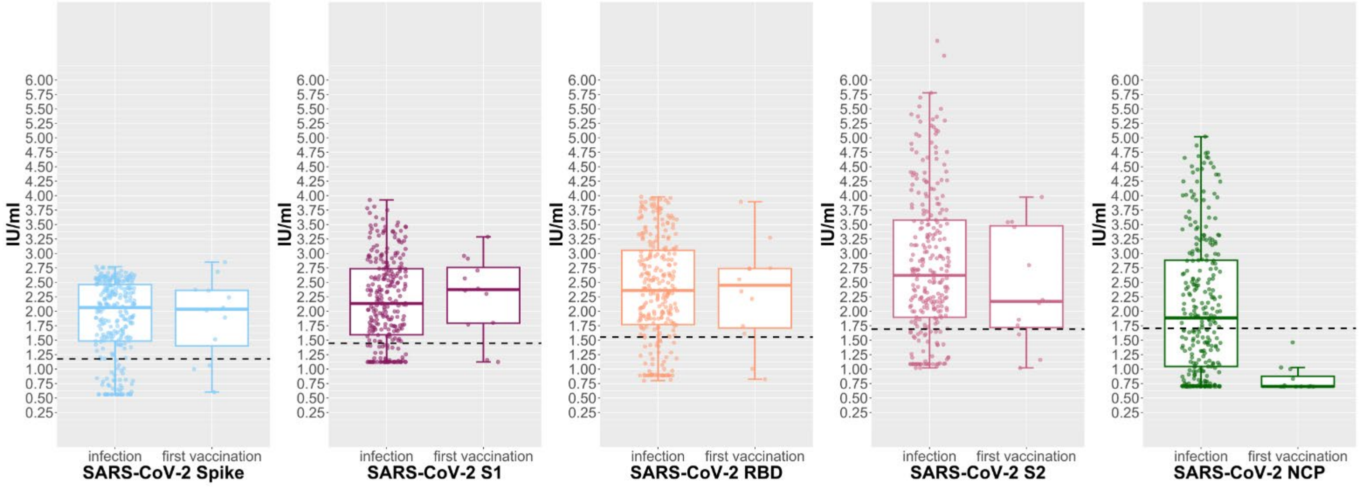

Figure S3. Antibody levels against the five SARS-CoV-2 antigens in international units. The left boxplots represent individuals with previous SARS-CoV-2 infection and the right boxplots represent individuals with vaccination as their first immunizing event.

3.2.2. Immuno-reactive patterns against the SARS-CoV-2 domains in the immunized sample

Analogous to the immune-reactive patterns in infected and non-infected persons, we generated these patterns of antibody formation against the five SARS-CoV-2 domains for persons with infection or with vaccination as first immunization event. Most infected individuals (n = 150) showed an antibody response to all of the SARS-CoV-2 antigens (Figure S4). The second most frequent pattern is a response to all antigens except the nucleocapsid protein. This pattern was found in 40 infected and seven vaccinated individuals. 32 infected and one vaccinated person did not develop any antibodies. 11 infected individuals reacted only to the full spike, the RBD and the S2 domain. Another pattern is the response to all antigens except for the full spike antigen, found in six individuals with a positive PCR result. A positive antibody response to all antigens except the S1 protein is seen in five infected individuals. A total of 26 individuals developed other patterns. The formation of antibodies against any domain always involved RBD and S2 domains, making them to promising targets for specific therapeutic monoclonal antibodies. In contrast, S1 and NCP antibodies are missing in three of the abundant patterns observed, underlining lower antigenicity due to the high variability of these domains.

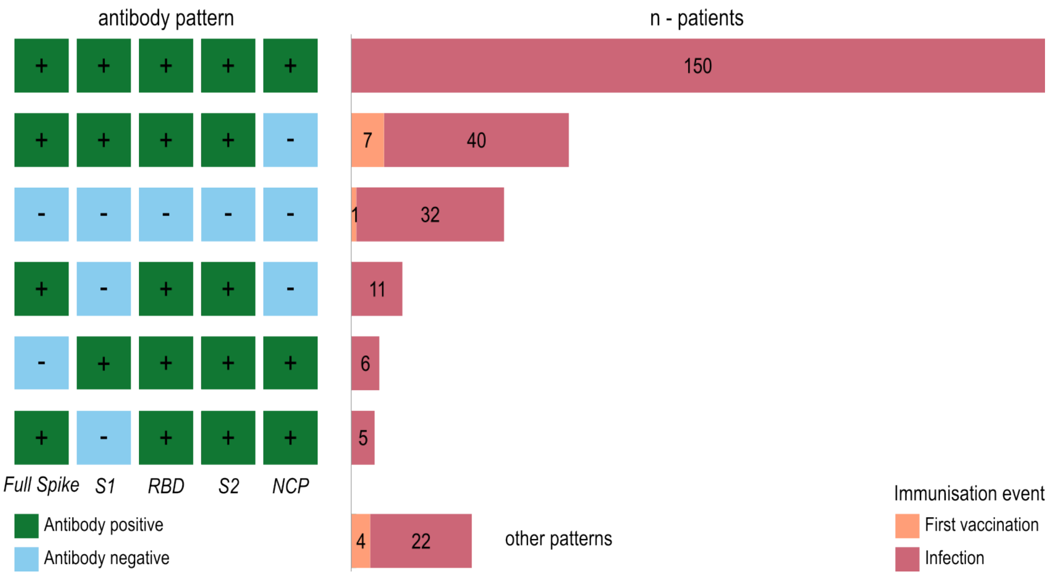

Figure S4. Antibody pattern in the 278 individuals of the immunized sample against the SARS-CoV-2 domains full spike, S1, receptor-binding domain (RBD), S2, and nucleocapsid protein. On the left side, the SARS-CoV-2 immuno-pattern is shown, where '+' and '−'signs indicate formation or absence of the respective antigen. The right side shows the corresponding number of individuals, separated for persons with a previous SARS-CoV-2 infection and those with vaccination as their first immunizing event. Only patterns present in at least four individuals are shown.

The number of detectable antibodies in the patterns differed between the infected and vaccinated groups. In the former, the median number of antibodies was five, in the latter four (Figure S5). In the infected group, a noticeable number of individuals did not develop any antibodies (indicated by the red circle in Figure S5). A patient specific response toward the virus is seen. However, a proportion of patients (12%) did not form specific antibodies according to the results.

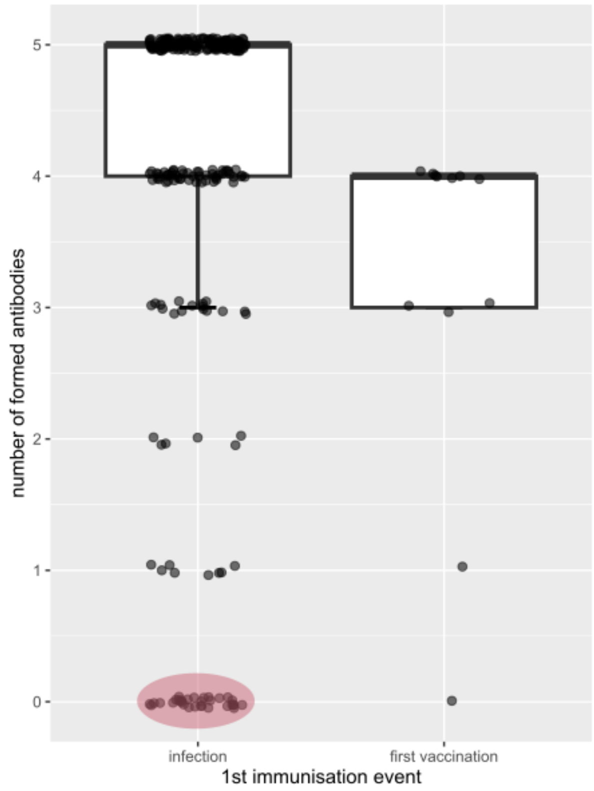

Figure S5. Number of antibodies formed against the five SARS-CoV-2 antigens full spike, S1, RBD, S2, NCP. The left boxplot represent the individuals with a previous SARS-CoV-2 infection and the right boxplot the individuals with a vaccination as their first immunizing event.

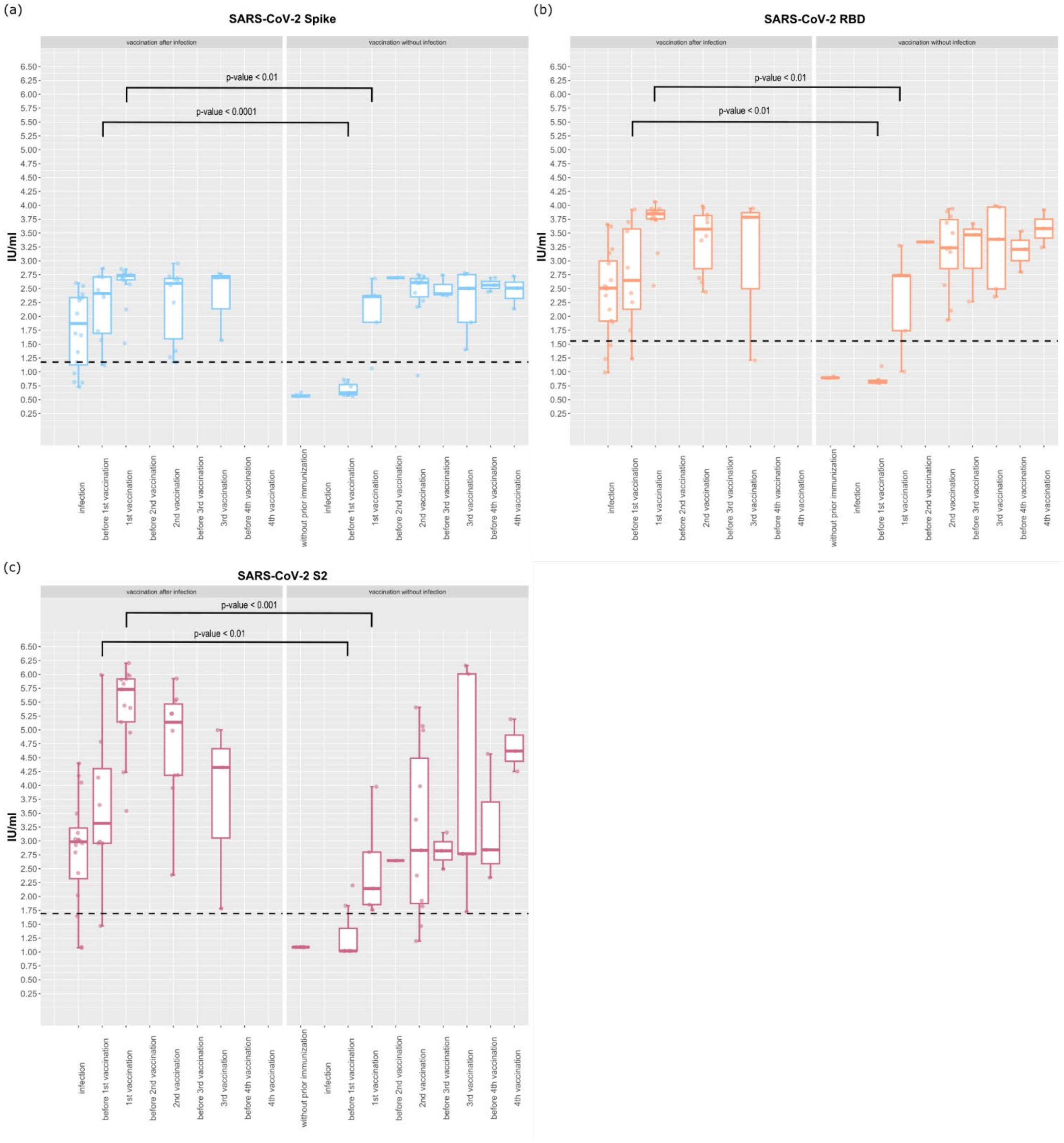

Figure S6. Longitudinal development of the antibody levels against (**a**) SARS-CoV-2 full spike domain, (**b**) RBD and (**c**) S2 domain. The boxplots on the left side indicate the antibody levels of individuals with previous SARS-CoV-2 infection and subsequent vaccination. The box-plots on the right side indicate individuals who were only vaccinated. The dashed lines mark the detection cut-offs, which differ for each domain.

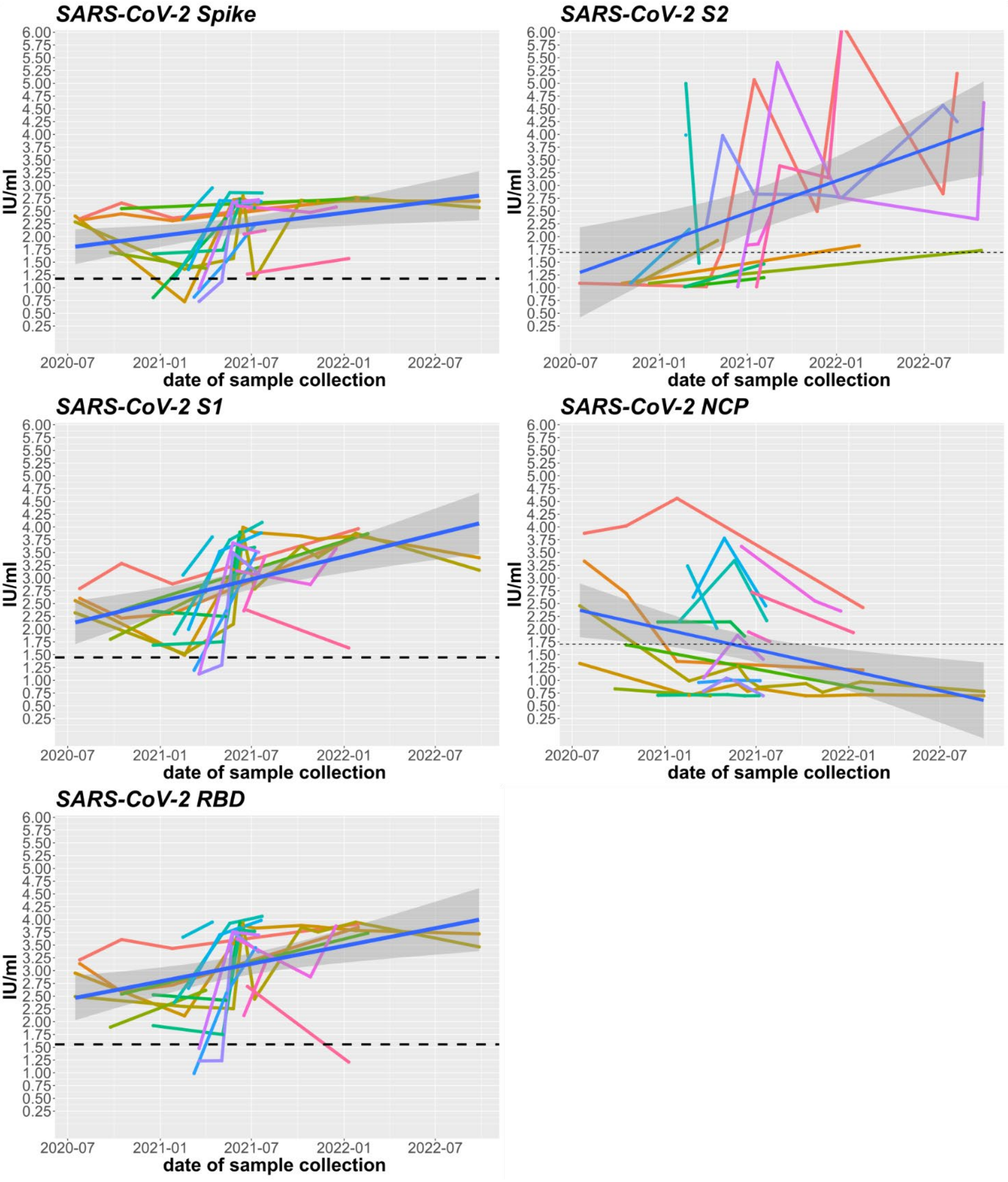

Figure S7. Longitudinal development of the antibody levels against each SARS-CoV-2 antigen for individuals infected prior to vaccination. Each colour represents to one person.

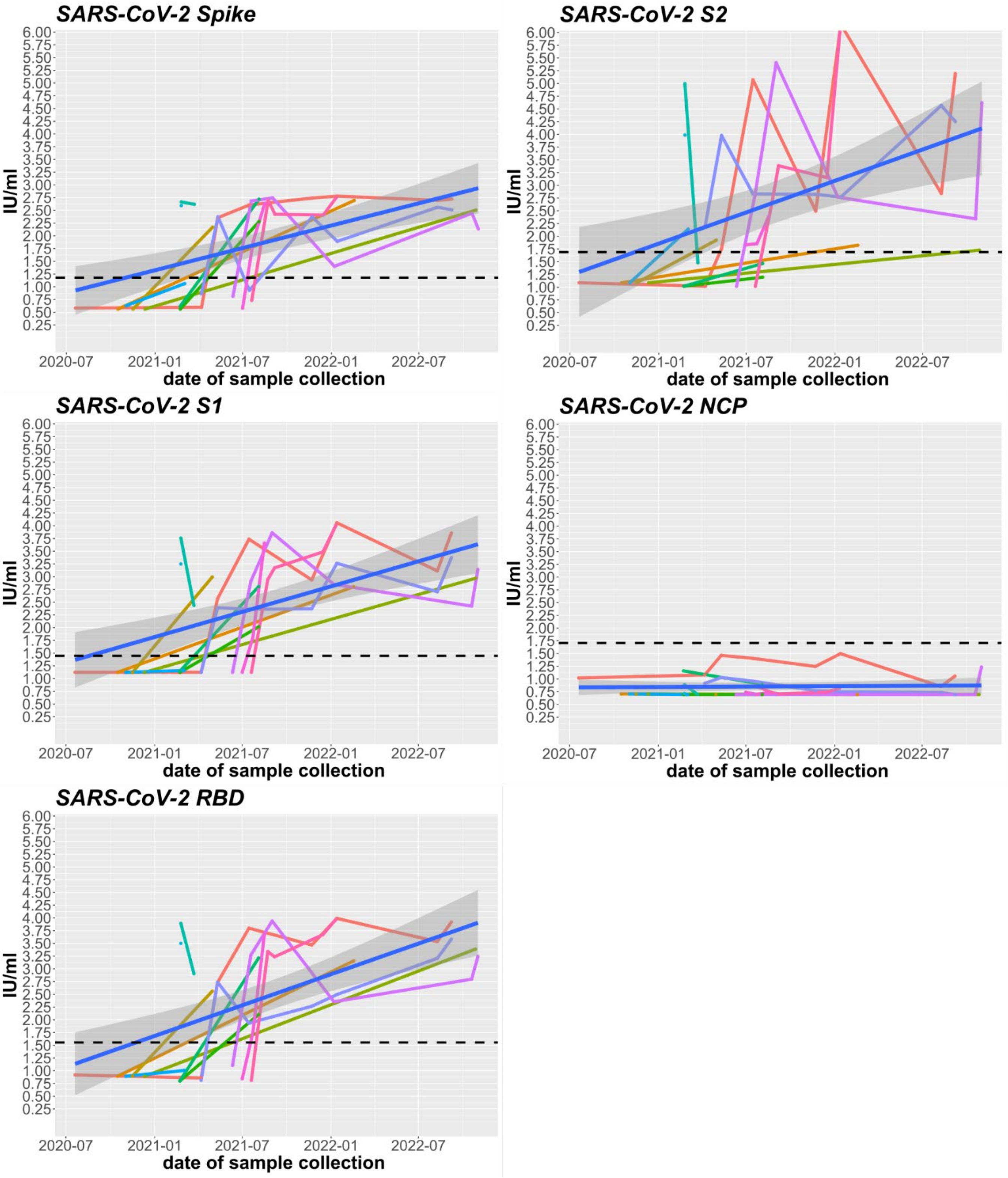

Figure S8. Longitudinal development of antibody levels to each of the SARS-CoV-2 antigens for individuals non infected prior to vaccination. Each colour represents to one person.
